# Adequacy to immunosuppression management guidelines in kidney transplant recipients with severe COVID-19 pneumonia: a practice survey

**DOI:** 10.3389/frtra.2024.1305152

**Published:** 2024-03-12

**Authors:** Amélie Jacq, Christelle Auvray, Mathieu Blot, Belaïd Bouhemad, Alice Casenaz, Baptiste Lamarthée, Mathieu Legendre, Jean-Pierre Quenot, Gilbert Zanetta, Claire Tinel

**Affiliations:** ^1^ Department of Nephrology and Kidney Transplantation, Dijon University Hospital, Dijon, France; ^2^ Department of Virology, Dijon University Hospital, Dijon, France; ^3^ Department of Infectious Diseases, Dijon University Hospital, Dijon, France; ^4^ Anesthesia and Intensive Care Department, Dijon University Hospital, Dijon, France; ^5^ TAI-IT Department, Inserm UMR Right, Université de Franche Comté, EFS BFC, Besançon, France; ^6^ Medical Intensive Care Department, Dijon University Hospital, Dijon, France

**Keywords:** kidney transplant recipients, COVID-19, medical practices survey, vaccination, intensive care unit

## Abstract

**Introduction:**

Coronavirus disease 2019 (COVID-19) poses an important risk of morbidity and of mortality, in patients after solid organ transplantation. Recommendations have been issued by various transplantation societies at the national and European level to manage the immunosuppressive (IS) regimen upon admission to intensive care unit (ICU).

**Method:**

The aim of this study was to evaluate the adequacy of IS regimen minimization strategy in kidney transplant recipients hospitalized in an ICU for severe COVID-19, in relation to the issued recommendations.

**Results:**

The immunosuppressive therapy was minimized in all patients, with respectively 63% and 59% of the patients meeting the local and european recommendations upon admission. During ICU stay, IS was further tapered leading to 85% (local) and 78% (european) adequacy, relative to the guidelines. The most frequent deviation was the lack of complete withdrawal of mycophenolic acid (22%). Nevertheless, the adequacy/inadequacy status was not associated to the ICU- or one-year-mortality.

**Discussion:**

In this single-center cohort, the only variable associated with a reduction in mortality was vaccination, emphasizing that the key issue is immunization prior to infection, not restoration of immunity during ICU stay.

## 1 Introduction

Coronavirus disease 2019 (COVID-19) is an infectious disease caused by the SARS-CoV-2 virus and was declared a global pandemic by the World Health Organization since 2020. The severe form of the disease is characterized by an acute respiratory distress syndrome (ARDS). COVID-19 is associated with a high risk of morbidity and mortality in the general population, and particularly in fragile patients such as solid organ transplant (SOT) recipients (1). Their higher risk of infection relies on their immunosuppressive treatment, but also because of their underlying comorbidities and frequent contact with the healthcare system. In addition, clinical manifestations may be different and more severe than in non-solid organ transplant patients. Their immune defense mechanisms, particularly the T lymphocyte response, are impaired, reducing viral clearance. Half of kidney transplant recipients (KTRs) still have a sustained viral load at 30 days from diagnosis, and their polymerase-chain receptor (PCR) molecular test remains positive up to 60 days after the onset of symptoms, compared with an average of 25 days in immunocompetent patients (2). Studies were rapidly carried out in this vulnerable population to investigate clinical manifestations and severity. The prognosis is more severe for KTRs, with a seven-fold increase in the risk of death compared with the general population. The occurrence of acute renal failure alone is an independent risk factor for mortality in this transplant population (3).

As of December 2020, the development of effective messenger RNA (mRNA) vaccines marked a turning point in the prevention of COVID-19 disease. However, this strategy has limited efficacy in KTRs, due to a lower vaccine response compared to immunocompetent patients. The seroconversion rate is largely influenced in part by patients’ immunosuppressive treatment, particularly with the use of mycophenolate mofetil (MMF), belatacept and rituximab, but it is also dependent on the time since transplantation, the presence of any diabetes or the level of graft function (4). For example, the seroconversion rate in transplant recipients was only 10% after the first vaccination dose, rising to 50% after the second dose (5). Empirically, the number of doses was increased to 3 and then 4 in this population at high risk of severe forms of the disease. Clinical studies have confirmed that a 4th dose significantly increases antibody levels in over half of KTRs (6).

For patients with insufficient antibody levels despite an appropriate vaccination regimen, neutralizing anti-SARS-CoV-2 monoclonal antibodies were then developed for prophylactic use. Several trials have demonstrated that these monoclonal antibodies accelerate viral clearance and significantly limit the number of hospitalizations or the unfavorable evolution of COVID-19 (7, 8). The European Medicines Agency, followed by France's Haute Autorité de Santé, rapidly authorized early access for patients at risk of severe COVID-19, including KTRs. These antibodies target a specific part of the virus known as the receptor binding domain, located on the N-terminal S1 subunit, thereby inhibiting binding and fusion of the virus with the body's healthy cells. Unfortunately, this part of the virus is poorly conserved and differs from one variant to another, rendering this therapy ineffective. Specific treatments have also been developed to either inhibit viral proliferation, or play an immunomodulatory role by inhibiting the hyperinflammatory phase causing ARDS. Notably, several trials are underway to develop new monoclonal antibodies (9). Anti-inflammatories such as dexamethasone (10) and anti-IL-6 tocilizumab (11) have been widely used, as have specific treatments such as remdesivir (a viral RNA polymerase inhibitor) (12). Corticosteroid therapy with dexamethasone has been recommended as the first line of treatment by all learned societies (13).

In KTRs, the question has therefore arisen as to how to adjust immunosuppressive therapy in the event of COVID-19 disease, in order to facilitate viral clearance while limiting the risk of rejection and the development of antibodies directed against the graft. In the case of other opportunistic infections, the “Kidney Disease: Improving Global Outcome” (KDIGO) guidelines (14) recommend reducing or even temporarily suspending immunosuppression. This would allow the development of specific immunity (15). In addition, immunosuppressive therapy must be adapted to the concomitant use of anti-COVID-19 therapies, which can induce drug interactions. This is the case with the nirmatrelvir/ritonavir (16) combination, which requires suspension of calcineurin inhibitors (CNI) due to a major risk of overdosage. A group of experts from the European Renal Association—European Dialysis and Transplantation Association (ERA-EDTA) (17) has described different strategies for reducing immunosuppression, depending on the degree of severity of COVID-19. The discontinuation of all immunosuppressive drugs except steroids was recommended for severe cases. However, continuing with low-dose CNI was to be considered for patients with higher risk of rejection (<1 year after transplantation and/or highly immunized).

In France, the Société Francophone de Transplantation (SFT) proposed in April 2020 a practical guide for the management of adult solid organ transplant patients affected by COVID-19 (https://www.transplantation-francophone.org/images/public/COVID19_et_transplantees_d_organes_solides_Guide_pratiquev1_SFT_SFNDT_SP.pdf). This guide suggests that the patient's referring transplant department should be informed and assist in the overall therapeutic management of the patient. A course of action is provided for symptomatic outpatient COVID-19 disease, symptomatic inpatient disease with no signs of severity, and severe COVID-19 disease with respiratory distress. For the latter, treatment with MMF and mammalian target of rapamycin (m-TOR) inhibitors must be stopped on admission. Temporary discontinuation of calcineurin inhibitors should be discussed on a case-by-case basis.

Despite numerous studies of COVID-19 and renal transplantation, the impact of immunosuppression is unclear. It may protect against the cytokine storm produced by COVID-19 infection, but its cessation or reduction may restore the immune system to fight the infection. There are few studies on the management of immunosuppression in severe COVID-19 with intensive care unit (ICU) admission, or on the outcome of patients and their renal function. In this context, we carried out a practice survey to assess the appropriateness of the immunosuppressant minimization strategy for KTRs hospitalized in ICU for COVID-19 between March 2020 and March 2023 at the Centre Hospitalier Universitaire (CHU) de Dijon, with the SFT recommendations.

## 2 Material and methods

### 2.1 Study population

This was a monocentric retrospective study carried out in the intensive care and intensive medicine departments of Dijon University Hospital. Inclusion criteria were: (i) renal transplant patients followed at Dijon University Hospital, (ii) hospitalized in one of these two intensive care units between March 1, 2020 and March 31, 2023 (iii) with severe SARS-CoV-2 infection.

**Figure 1:**
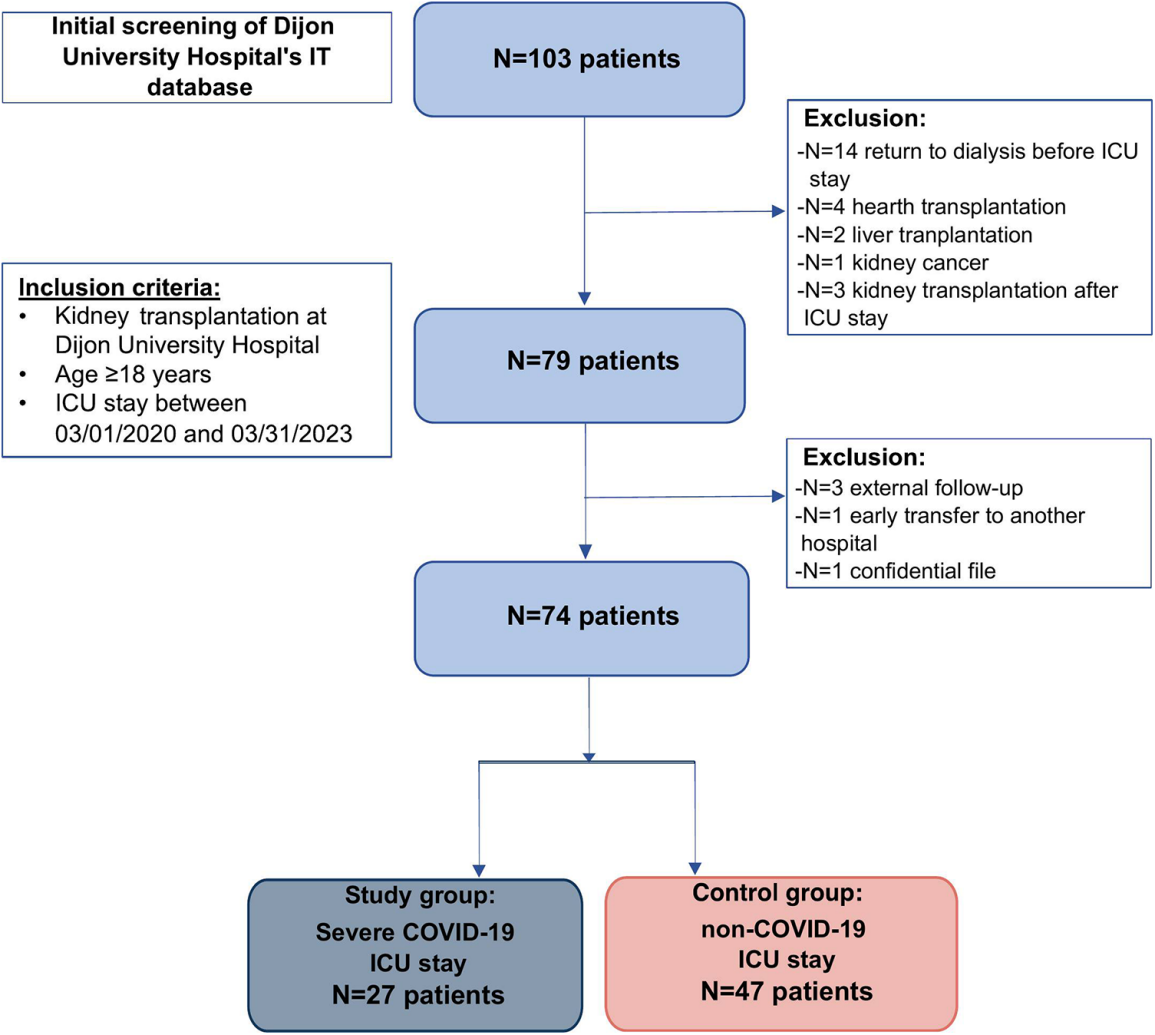
Flowchart of the study.

The flow chart (Figure 1) shows the patient selection and inclusion strategy. Over the period of interest, the IT system (Programme de Médicalisation des Systèmes d'Information) identified 103 renal transplant patients hospitalized in the ICU. A careful review of the chart excluded *N* = 24 patients who did not meet the inclusion criteria (return to dialysis before ICU stay *N* = 14, non-renal SOT *N* = 6, ICU stay prior to kidney transplantation *N* = 3, no transplantation *N* = 1). A total of 79 KTR were admitted in ICU during the study period. To limit missing data, three patients who were not transplanted or followed at Dijon University Hospital and one confidential file were excluded. Additionnally, one patient was excluded for early transfer to another French University Hospital, due to saturation of local intensive care services at that time. Full data were available for 74 KTR who were included in this study, of which 27 patients were hospitalized in ICU for severe COVID-19 disease (Study group), and 47 patients were hospitalized for another disease (Control group). In this non-COVID group, the main reasons for hospitalization were as follows: post-operative intensive care (*N* = 17, 36%), non-COVID-related ARDS (*N* = 8, 17%), shock (*N* = 7, 15%), stroke (*N* = 7, 15%), heart failure (*N* = 2), cancer-related intensive care (*N* = 2), other (*N* = 4).

### 2.2 Clinico-biological data

Clinico-biological data during hospitalization in the ICU were collected retrospectively from patients’ paper or computerized records. Donor data were collected using the Agence de Biomédecine's Cristal software. If the patient has received more than one kidney transplant (retransplantation), the transplant during which the COVID-19 disease occurred is referred to as an “active transplant”. The calculation of time spent on dialysis then takes into account only the time spent on dialysis immediately prior to the active transplant.

Patients who were negative to HLA antibody before transplantation and received their first allograft are considered “low risk” patients. High immunological risk was defined as a second kidney transplant and/or the presence of historical or day 0 donor-specific antibody (DSA) and/or an Panel reactive antibody (PRA) ≥85%. Donor types include living donors (LD), donation after brain death donors (DBD) and donation after circulatory death (DCD). DBD donors are classified into extended-criteria donors (ECD) and standard-criteria donors (SCD). ECD donors correspond to all donors ages over 60 years, or donors aged between 50 and 59 presenting two of the following criteria: history of arterial hypertension, death from cerebrovascular causes, creatinine >1.5 mg/dl. Delayed graft function (DGF) was defined as the need for dialysis within seven days of renal transplantation. Renal function was assessed by creatinine and estimated glomerular filtration rate (eGFR) calculated by the CKD-EPI method at the following predefined times: 6 months and 3 months before ICU admission (D0), at D0, then at D5, D15, D30, 6 months and 1 year after. 24 h proteinuria was assessed before admission to the ICU, and at 3, 6 and 12 months after admission. SARS-CoV-2 infection was confirmed by PCR testing. COVID-19 disease was considered severe if only one of the following criteria occurred: oxygen saturation below 90% in room air, respiratory rate above 30 breaths per minute, signs of acute respiratory distress (use of accessory muscles, inability to form a complete sentence). COVID-19 disease with critical status is defined by the criteria of acute respiratory distress syndrome, a septic state, septic shock or other problems normally requiring vital care, such as mechanical ventilation (invasive or non-invasive) or administration of vasopressors.

### 2.3 Immunosuppressive regimen

The intensity of immunosuppressive treatment on arrival in the ICU and its evolution during the stay were assessed by: (i) the mean of the last 3 residual rates (T0) and then the T0 for CNI and imTOR, (ii) the daily dosage for antimetabolites (MMF or azathioprine) and corticosteroids. Management of immunosuppression was assessed according to whether or not the patient sought advice from the transplant team, and whether or not any immunosuppressive treatment was minimized (dosage reduced or stopped). Overdosage was defined, independently of post-transplant time, as a residual tacrolimus level >10 ng/ml or a residual cyclosporine level >200 ng/ml or a sirolimus/everolimus level >10 ng/ml.

### 2.4 Immunological status against SARS-Cov-2

Patients’ immunological status against SARS-CoV-2 at D0 was estimated on the basis of the following criteria: presence or absence of vaccination, number of vaccination doses received, serology (anti-SARS-CoV-2 IgG) prior to the possible administration of monoclonal antibodies, administration of anti-SARS-CoV-2 monoclonal antibodies, their specificity and the date of the last injection. A patient was considered as responder to vaccination if his or her anti-Spike IgG level was greater than 264 BAU/ml (conversion to AU/ml with a multiplication factor of 0.142 where applicable) (18). A patient was considered theoretically protected against a severe form of COVID-19 disease if he responded to vaccination with a last dose within 6 months, or if he received monoclonal antibodies as pre-exposure prophylaxis. It should be noted that it was not possible to individually define the sensitivity of the SARS-CoV-2 variant identified to the last monoclonal antibodies received.

### 2.5 Clinical endpoints

The primary endpoint was the adequacy of the immunosuppression minimization strategy to the recommendations of the French Society of Transplantation for the management of renal transplant patients admitted to the ICU (https://www.transplantation-francophone.org/images/public/COVID19_et_transplantees_d_organes_solides_Guide_pratiquev1_SFT_SFNDT_SP.pdf) or to the European guidelines of the European Renal Association (17). Secondary endpoints included patient outcome (survival), renal function outcome in the year following the ICU stay (estimated GFR, 24 h proteinuria) and graft outcome (occurrence of rejection, graft loss).

### 2.6 Statistical analysis

Quantitative variables are expressed by their mean and standard deviation, or by their median and interquartile range (IQR). Qualitative data are expressed as numbers and percentages. Patient characteristics between Study and Control groups were compared using the Fisher's exact test for percentages and the Kruskall-Wallis test for medians. Glomerular filtration rate (GFR) and trough levels (T0) values were compared by a non-parametric test for paired values (Wilcoxon matched-pairs signed rank test). Survival analyses were performed using Kaplan-Meier curves and the Cox proportional hazards regression model. A value of *P* < 0.05 was considered significant. Statistical analyses were performed in the RStudio environment (version 2022.12.0 + 353) and using GraphPad Prism software (version 10.0.2 [171]).

## 3 Results

### 3.1 Characteristics of the population at renal transplantation

During the interest period, 27 kidney transplant recipients followed at Dijon University Hospital with a functional graft were hospitalized in the intensive care units for the management of a severe form of COVID-19 (Study group). Patient characteristics and their medical background at the time of renal transplantation are presented in Table 1. The median age at transplantation was 62 years (IQR: 51.8–68.7), with a majority of men (78%). The main cause of chronic end-stage renal disease (41%) was hereditary or congenital nephropathy (including polycystic kidney disease), followed by primary glomerulopathy in 19% and secondary glomerulopathy in 11% of cases. The majority of patients were already on dialysis (89%), for a median duration of 39 months.

**Table 1:** Patients characteristics at transplantation in the study group.

Variables	*N* = 27
Age at transplantation (years), median (IQR)	62 (16,5)
Sex (male), *n* (%)	21 (78)
Nephropathy, *n* (%):	
Hereditary or congenital	11 (41)
Primary glomerulonephritis	5 (19)
Secondary glomerulonephritis	3 (11)
Diabetic nephropathy	2 (7)
Nephroangiosclerosis	2 (7)
Interstitial nephropathy	2 (7)
Unknown	2 (7)
Preemptive transplantation, *n* (%)	3 (11%)
Dialysis time (months), median (IQR)	39 (33)

IQR, interquartile range.

### 3.2 Transplant characteristics

Table 2 shows the main characteristics of renal transplantation. Most patients were receiving their first transplantation with only 15% of retransplantation. The median panel reactive antibody (PRA) was 32%, and two were considered as hyperimmunized patients. All patient were negative to HLA-DSA anytime prior to- or at the time of renal transplantation. According to the defined criteria, 81.5% were considered low-risk patients. Deceased donors encounted for 93% of cases with a majority of expanded criteria donors (ECD). Induction therapy consisted in basiliximab (56%) or Thymoglobulins (44%). In the immediate post-transplant period, maintenance therapy included a combination of steroids, MMF and CNI for 25/27 patients. Ciclosporin was used in 69% of cases, and tacrolimus in 31%.

**Table 2:** Transplant characteristics in the study group.

Variables	*N* = 27
Transplantation rank >1, *n* (%)	4 (15)
PRA >85%, *n* (%)	2 (7,4)
Donor type, *n* (%)	
LD	2 (7,4)
DBD SCD	10 (37,1)
DBD ECD	14 (51,8)
DCD Maastricht 3	1 (3,7)
Cold ischemia time (hours), median (IQR)	14,7 (8,9)
DGF, *n* (%)	1 (4)
Induction therapy, *n* (%)	
Thymoglobulins, *n* (%)	12 (44)
Basiliximab, *n* (%)	15 (56)
Maintenance therapy, *n* (%)	
Steroids, *n* (%)	27 (100)
Calcineurin inhibitors, n(%)	26 (96)
Ciclosporin, *n* (%)	18 (69)
Tacrolimus, *n* (%)	8 (31)
Antimetabolites, *n* (%)	27 (100)
Mycophénolate mofétil, *n* (%)	26 (96)
Azathioprine, *n* (%)	1 (4)
mTOR inhibitors, *n* (%)	1 (4)

DBD, donation after brain death; DCD, donation avec cardiac arrest; DGF, delayed graft function; ECD, expanded criteria donor; IQR, interquartile range; LD, living donor; mTOR, mammalian target of rapamycin; SCD, standard criteria donor.

### 3.3 Risk factors for COVID-19 infection

The risk factors for severe SARS-CoV-2 infection in the Study and Control groups are presented in Table 3. In the Study group, the median age at ICU admission was 66 years with a median post-transplant time of 3 years and 8 months (range: 1 month-26 years). A high-risk cardiovascular profile was common, with 26% of diabetes mellitus, 93% of high blood pressure and 22% of obesity. Only two patients were active smokers. None had a history of chronic obstructive pulmonary disease. Seven patients (26%) received an immunosuppressive therapy prior to the active renal transplantation, for their previous transplantation (*N* = 4) or for the treatment of their primary kidney disease (*N* = 3). Additionnaly, two patients received steroid pulses for the treatment of a biopsy-proven acute rejection (BPAR). For all these risk factors for severe COVID-19 disease, there were no significant differences between the Study and the Control group.

**Table 3:** Risk factors for severe COVID-19.

Variables	COVID group	Control group	*P* value
	*N* = 27	*N* = 47	
Conventional COVID19 risk factors			
Age at onset (years), median (IQR)	66 (13)	62 (13)	0.19
Diabetes mellitus, *n* (%)	7 (26)	15 (32)	0.79
High blood pressure, *n* (%)	25 (93)	44 (94)	>0.99
Active smoking, *n* (%)	3 (11)	4 (8)	0.70
BMI ≥30 kg/m2, *n* (%)	6 (22)	11 (23)	>0.99
Transplantation-associated risk factors *(excluding induction & maintenance therapy)*			
Time from KT (months), median (IQR)	45 (88)	82 (141)	0.17
Lymphocyte count (/ml), median (IQR)	1,330 (860)	1,020 (1,140)	0.32
Total IgG (g/L), median (IQR)	8.1 (2.8)	8.8 (3.7)	0.18
IS therapy prior to active KT, *n* (%)	7 (26)	7 (15)	0.36
Treated BPAR**, *n* (%)	2 (7)	0 (0)	0.13
Anti-SARS-Cov2 vaccination**, *n* (%)	17 (63)	26 (55)	0.63
1 dose, *n* (%)	2 (11.8)	2 (7,7)	
2 doses, *n* (%)	1 (5.9)	5 (19.2)	
3 doses, *n* (%)	11 (65)	14 (54)	
4 doses, *n* (%)	3 (17.6)	5 (19.2)	
Prophylactic monoclonal antibodies, *n* (%)	6 (22)	4 (9)	0.16
Casirivimab/imdevimab, *n* (%)	3 (11)	2 (4)	
Tixagevimab/cilgavimab, *n* (%)	2 (7)	1 (2)	
Casirivimab/imdevimab, then Tixagevimab/cilgavimab, *n* (%)	1 (4)	1 (2)	
Theoretical immunisation, *n* (%)	7 (26)	13 (28)	0.41
eGFR at M-3** (ml/min/L,73m^2^), median (IQR)	32 (18)	38 (32)	**0**.**04**

BMI, body mass index; BPAR, biopsy-proven acute rejection; eGFR, estimated glomerular filtration rate; IQR, interquartile range; IS, immunosuppressive; KT, kidney transplantation.

Bold values indicate a *P* value < 0.05.

** Prior the ICU admission.

Concerning anti-SARS-CoV-2 vaccination, 17 patients (63%) had received at least one dose prior to their infection, of whom 14 had received ≥3 doses. Six patients (22%) also received anti-SARS-CoV-2 monoclonal antibodies as pre-exposure prophylaxis. According to the defined criteria, 7 (26%) patients were considered as theoretically protected from a severe form of COVID-19. For all these immunological status for SARS-Cov-2, no significant difference was observed between the Study and the Control group. It should be noted however, that the eGFR 3 months prior to hospitalization was significantly lower in the Study group, with a well-established link between impaired renal function and impaired immune defenses.

### 3.4 Description of ICU care stay

Table 4 summarizes the characteristics of the ICU stay in the Study and Control groups. As expected, patients in the Study group have more severe characteristics, judging by the median length of stay (14 vs. 3 days, *P* < 0.0001), the use (*P* < 0.05) and median duration of mechanical ventilation (17 vs. 3 days, *P* < 0.0001), the use of prone positioning (*P* < 0.0001), and the use (*P* < 0.001) and median duration of vasopressors (8 vs. 2 days, *P* < 0.01). Compared with their baseline eGFR three months earlier, acute kidney injury occured in 100% of COVID patients upon admission while in 45% of KTR in the non-COVID group (*P* < 0.0001). Renal replacement therapy (RRT) was required for 59% of patients in the Study group and in 36% of the control group (*P* = 0.09), with a longer duration for EER (respectively 5 vs. 3 days, *P* < 0.05). Coinfection occurred more frequently in case of an initial COVID-19 disease (85% vs. 34%, *P* < 0.0001), most often with bacterial respiratory tract infection (70% vs. 17%, *P* < 0.05) bacteriemia (41% vs. 13%, *P* < 0.001), or urinary tract infection (30% vs. 6%, *P* < 0.05). Five patients had concomitant cytomegalovirus (CMV) viremia in the Study group and two in the Control group (*P* = 0.09). In addition to corticosteroid therapy, seven patients received COVID-19-specific treatment: six received anti-IL6 monoclonal antibodies (tocilizumab) and one patient received sotrovimab. No patient received remdesivir therapy, as it indication according to local and international authorities is limited to non-severe COVID-19 disease.

**Table 4:** Characteristics, treatment and outcome during ICU stay.

Variables	COVID group	Control group	*P* value
	*N* = 27	*N* = 47	
Length of stay (days), median (IQR)	14 (26.5)	3 (5.5)	**<0**.**0001**
Mechanical ventilation, *n* (%)	21 (78)	23 (47)	**<0**.**05**
Duration (days), median (IQR)	17 (27)	3 (8)	**<0**.**0001**
Prone position, *n* (%)	17 (81)	1 (4)	**<0**.**0001**
Vasopressor, *n* (%)	21 (78)	17 (35)	**<0**.**001**
Duration (days), median (IQR)	8 (11)	2 (3)	**<0**.**01**
Kidney function			
eGFR (ml/min/1,73m^2^) at admission, median (IQR)	26 (22,5)	34 (33)	0.17
AKIN classification, *n* (%)			
No AKI	0 (0)	21 (45)	**<0**.**0001**
Stade 1	3 (11)	5 (11)	>0.99
Stade 2	2 (7)	4 (9)	>0.99
Stade 3	16 (59)	17 (36)	0.09
Duration of dialysis (days), median (IQR)	5 (10,2)	3 (2)	**<0**.**05**
Co-infections, *n* (%)	23 (85)	16 (34)	**<0**.**0001**
Bacteriemia	11 (41)	6 (13)	**<0**.**001**
Pyelonephritis	8 (30)	3 (6)	**<0**.**05**
Bacterial pneumonia	19 (70)	8 (17)	**<0**.**0001**
CMV infection	5 (19)	2 (4)	0.09
Specific COVID-19 therapy, *n* (%)	7 (26)	–	–
Mortality, *n* (%)	12 (44)	9 (19)	**<0**.**05**

AKIN, acute kidney injury; CMV, cytomegalovirus; eGFR, estimated glomerular filtration rate; IQR, interquartile range.

Bold values indicate a *P* value < 0.05.

**Figure 2:**
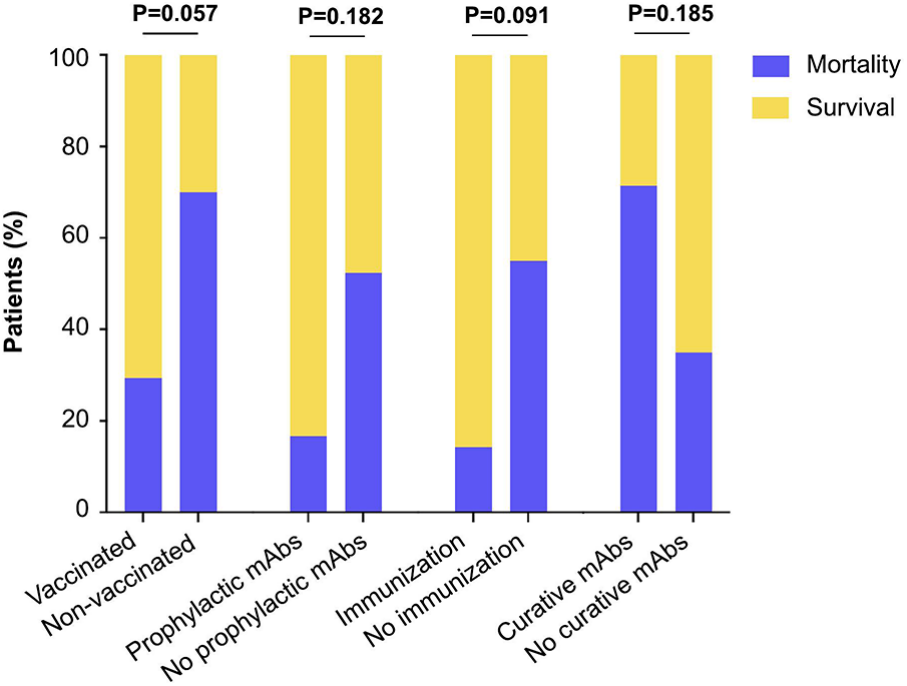
Mortality and survival according to different parameters related to COVID-19 prevention/treatments. The proportion of patients is shown in the bar graph. Fisher exact test *P* values are shown.

In all, the ICU mortality was significantly higher in the COVID group as compared to the non-COVID group (44% vs. 19%, *P* < 0.05). In the COVID group, ICU mortality rates were 29% vs. 70% (*P* = 0.057) in vaccinated patients as compared to non vaccinated patients respectively, 17% vs. 52% (*P* = 0.1882) in patients receiving monoclonal antibodies for prophylaxis or not, 14% vs. 55% (*P* = 0.091) in patients defined as theoretically immunized or not, and 71% vs. 35% (*P* = 0.185) in patients receiving curative monoclonal antibodies or not (Figure 2).

### 3.5 Immunosuppression management and adequacy to local and European guidelines

**Figure 3:**
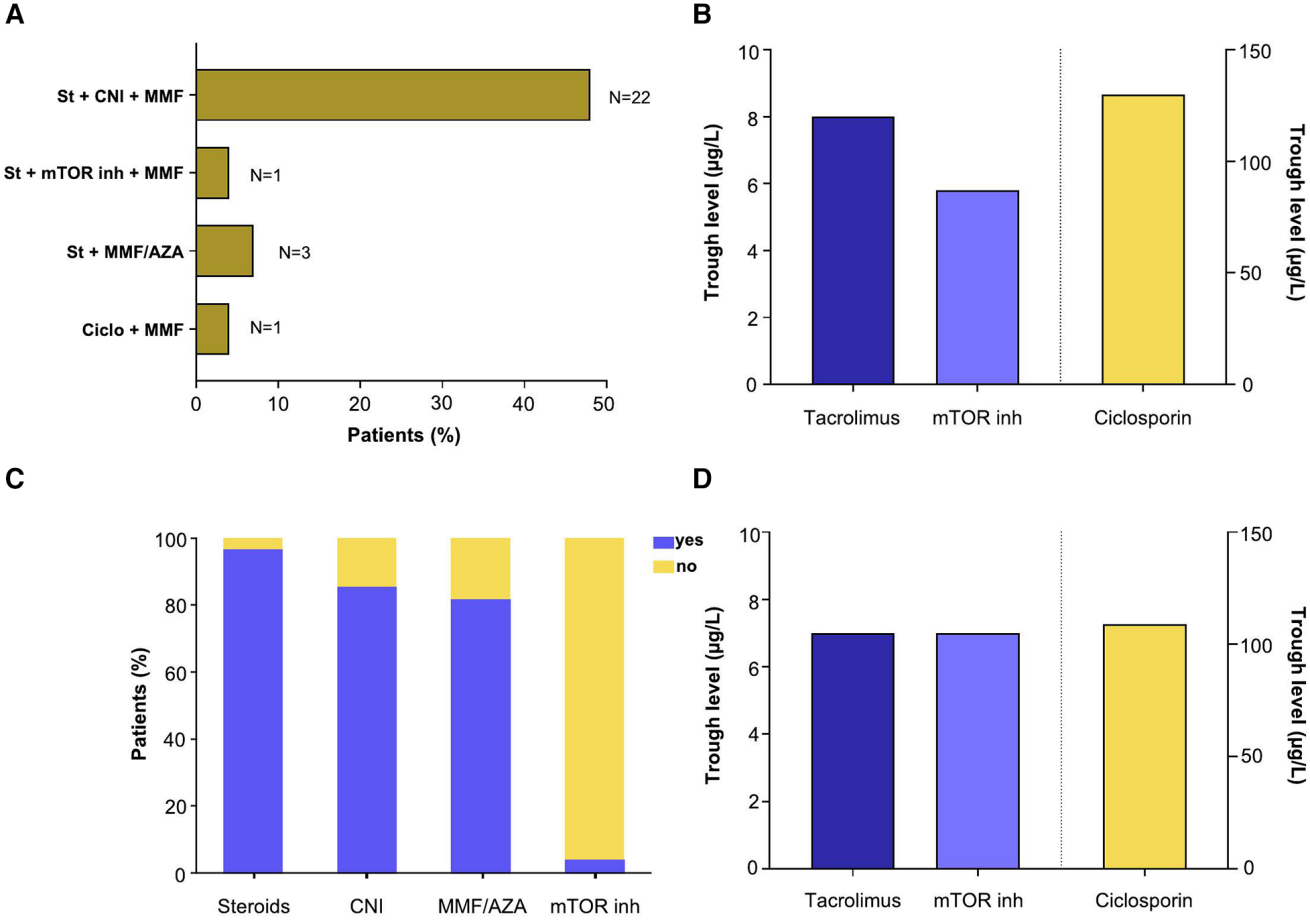
Maintenance immunosuppressive therapy. (**A**) Immunosuppressive combination received at diagnosis of SARS-CoV-2 infection. (**B**) Mean of last three trough levels prior to diagnosis of SARS-CoV2 infection. (**C**) Immunosuppressive drugs received on admission to intensive care unit. (**D**) Mean trough levels on admission to the ICU.

As the European and French recommendations for adapting the immunosuppressive (IS) regimen apply only to COVID-19 infection, the next results focus on the Study group. Figure 3A recapitulates the IS regimen patients were receiving at the time of COVID-19 diagnosis. The most frequent association (*N* = 22/27, 48%) was a triple therapy consisting of steroids, CNI and MMF. One patient received a combination of steroids, m-TOR inhibitors and MMF. Four patients received dual therapy combining steroids and MMF or azathioprine (*N* = 3) or ciclosporin and MMF (*N* = 1). The mean daily dose was 8 mg for steroids, 1,500 mg for MMF and 100 mg for azathioprine. For drugs with narrow therapeutic index, the mean ± SD of the last three trough levels (T0) before COVID-19 diagnosis was 8 ± 3 ng/ml for tacrolimus, 5.8 ± 1 ng/ml for the patient treated with m-TOR inhibitors and 130 ± 52 ng/ml for ciclosporin (Figure 3B). Figure 3C shows the percentage of patients treated by therapeutic class upon admission to ICU. While 100% of patients were receiving an antimetabolite (MMF or azathioprine) at diagnosis, five patients had their treatment stopped prior to ICU admission. Trough levels were not measured within 24 h after ICU admission for two patients on tacrolimus and seven patients on ciclosporin (i.e., 39% of patients). When trough levels were measured and despite acute kidney injury, no significant difference was observed upon admission as compared to the average of the last three T0s prior to diagnosis (tacrolimus: 7 vs. 8 ng/ml, *P*-value = 0.94; ciclosporin: 109 vs. 130 ng/ml, *P*-value = 0.22; Figure 3D). Overdosing was observed in three of the 15 patients (20%) with an available T0 on admission.

**Figure 4:**
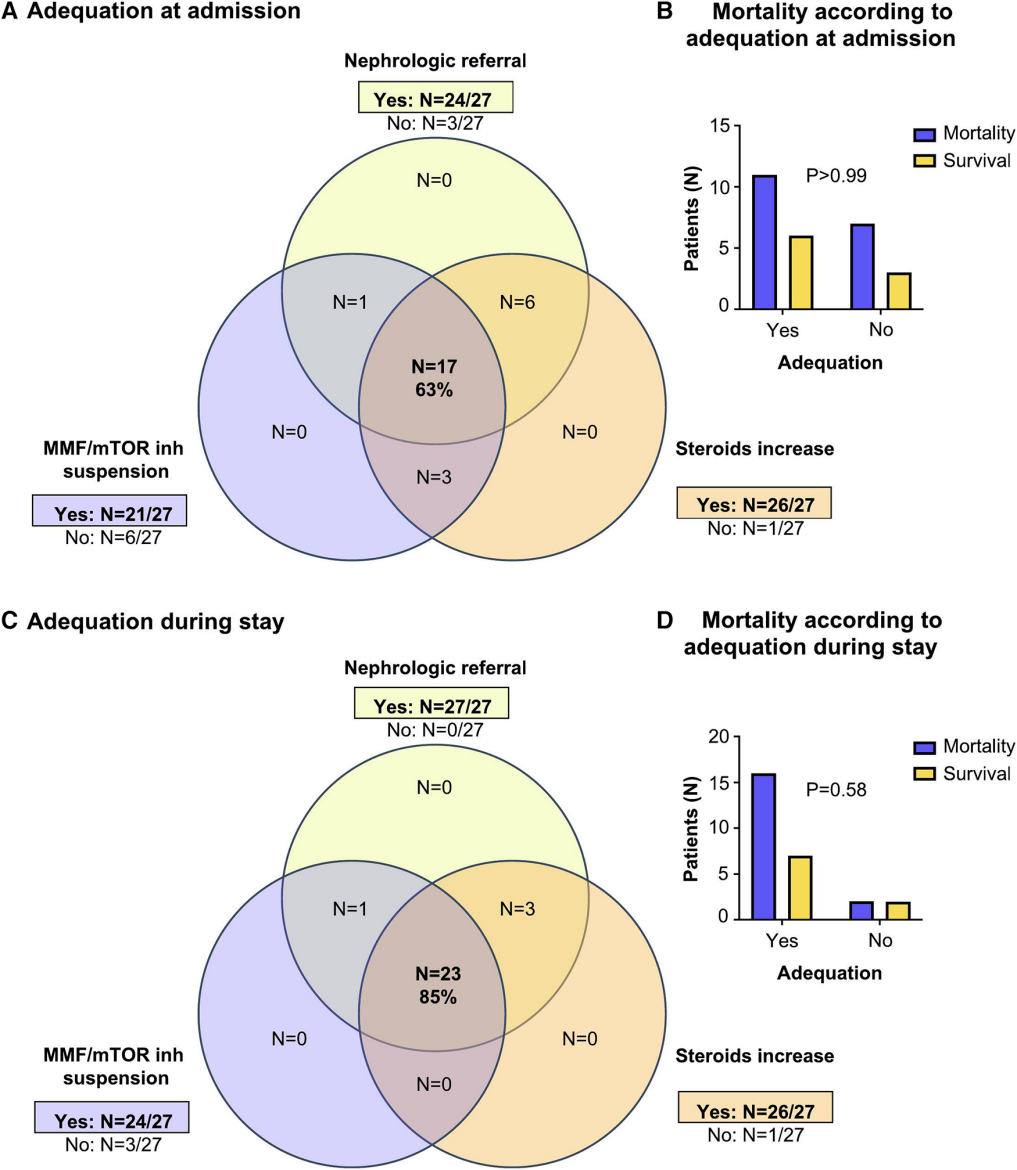
Adequacy between patient management and recommendations. (**A–D**) Local guidelines. (**A**) At admission: the Venn-diagram shows the number of patients in each group. (**B**) The number of surviving patients according to adequacy with recommendations at admission. The Fisher exact *p*-value is indicated. (**C**) During ICU stay: the Venn-diagram shows the number of patients in each group. (**D**) The number of surviving patients according to adequacy with recommendations during ICU stay. The Fisher exact test *p*-value is indicated. (**E–H**) European guidelines. (**E**) At admission: the Venn-diagram shows the number of patients in each group. (**F**) The number of surviving patients according to adequacy with recommendations at admission. The Fisher exact *p*-value is indicated. (**G**) During ICU stay: the Venn-diagram shows the number of patients in each group. (**H**) The number of surviving patients according to adequacy with recommendations during ICU stay. The Fisher exact test *p*-value is indicated.

With respect to local (French) guidelines, a nephrologic referral was sought for 24 patients (Figure 4A). The 3 patients who did not profit from nephrological referral, nevertheless benefitted from a minimization of their treatment. MMF was discontinued before admission in five patients, and MMF or mTOR inhibitors were further suspended upon admision in 16 other patients. For 3 patients, MMF dosage was reduced but not completely stopped. The remaining three patients had their dosage reduced but not completely stopped. With regard to corticosteroid therapy, the dosage was increased in 26 of the 27 patients. Altogether, 63% of patients met the local guidelines upon admission. Later during the ICU stay, further MMF withdrawal and nephrologic referral increased the recommendation adequacy up to 85% (Figure 4C). The one-year mortality rate was not significantly different in patients with an adequate management according to the local guidelines as compared to those considered as inadequately handled (at admission:65% vs. 70%, *P* > 0.99; during stay: 70% vs. 50%, *P* = 0.58, Figures 4B-D).

Considering the European guidelines, CNI treatment was adequately adjusted in 19 of the 23 patients on CNI (Figure 4E), with complete withdrawal in 17 patients, and continuing with low-dose CNI in 2 patients with higher risk of rejection (<1 year after transplantation). After a first decrease in dosage, CNI was further discontinued in 4 patients later during the stay (Figure 4G). Thus, the European recommendation (“all immunosuppressive drugs discontinuation except steroids”) was achieved in 59% of patients upon admission, and 78% during the stay. The only patient who did not benefit from a steroid increase was diagnosed as early as March 2020, right before/at the time of the recommendations issue. As before, no significant difference in the one-year mortality was observed in patients with an adequate (63%) or inadequate (73%) immunosuppressive management at admission (*P* = 0.69) or later during stay (*P* > 0.99, Figures 4F–H).

A comparative study in the IS regimen and their initial and final adjustments in KTR deceased in ICU or discharged from ICU is available in Supplementary Figure 1. Of note, MMF discontinuation was observed in 83% of KTR discharged after ICU as compared to 60% of deceased KTR in ICU, without reaching significance (*P* = 0.44).

### 3.6 Renal function and patient outcome

Twelve patients (44%) died during their ICU stay. Among those, RRT was required in 8 (67%) of patients. Withdrawal from EER was achieved in two patients. For all six patients (50%) who did not or no longer require dialysis, mean eGFR at 37.2 ml/min at the time of death.

**Figure 5:**
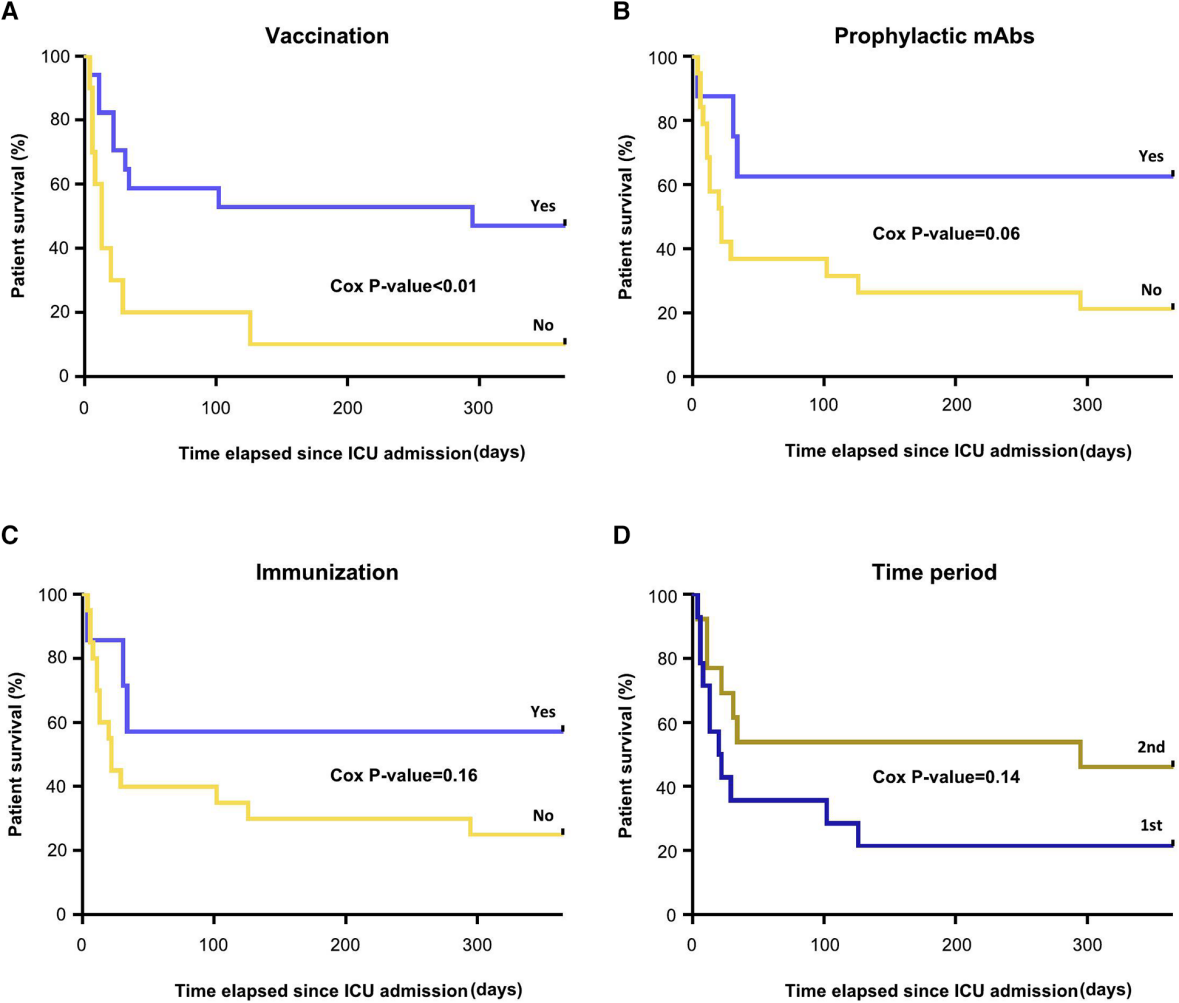
Patients survival during the first year of ICU stay according to (**A**) vaccination status (**B**) prophylactic mAbs (**C**) immunization and (**D**) time period. The Cox *P*-values are indicated.

Fifteen patients (56%) were discharged from ICU after their severe COVID-19. Patient and renal graft survival are presented in Table 5. Six patients died on general hospital wards shortly after their ICU discharge (mortality attributable to COVID-19), in a median time of 21 days. The one-year patient survival according to their immunological status or the time period is depicted in Figure 5. Vaccinated patients had a significantly better one-year survival than non-vaccinated patients (*P* < 0.01, Figure 5A). We observed a trend towards a better survival in patients who benefitted from prophylactic monoclonal antibodies (*P* = 0.06, Figure 5B) and in patients defined as immunized (*P* = 0.16, Figure 5C), without reaching statistical significance. In the first COVID-19 time period (March 2020–August 2021), survival seemed worse than in the 2nd time period (September 2021–March 2023, Figure 5D).

**Table 5:** Graft and patient outcomes.

Variables	Discharged from ICU	Discharged from ICU	*P* value
	*N* = 15 (56%)	*N* = 38 (81%)	
One-year BPAR, *n* (%)	0 (0)	0 (0)	–
Return to dialysis, *n* (%)	1 (9)	3 (8)	>0.99
COVID-related mortality after ICU discharge, *n* (%)	6 (43)	NA	–
Time (days) to COVID-related death after ICU, median (IQR)	21 (52)	NA	–
Overall one-year mortality, *n* (%)	18 (67)	18 (38)	**<0.05**

BPAR, biopsy-proven acute rejection; IQR, interquartile range; ICU, intensive care unit.

Bold values indicate a *P* value < 0.05.

Besides in the one-year period, graft failure occurred in one patient transplanted two months before COVID-19 infection, with return to hemodialysis 6 months after. Among surviving patients with a functioning graft, median eGFR was 39 ml/min at 3 months before ICU admission, and evolved as follows after COVID-19 infection: 69 ml/min at 1 month, 47 ml/min at 3 month, 46 ml/min at 6 months and 50 ml/min at 1 year. This unexpected apparent improvement in renal function is most likely reflecting the bias in renal function assessment when relying on serum creatinine in patients with severe amyotrophy. With respect to the IS regimen minimization during ICU stay, no acute rejection was noted in the one-year period.

Finally, the one-year death-censored graft survival was significantly lower in the COVID group than in the Control group (Supplementary Figure 2, Log-rank *P*-value <0.05), while the one-year mortality rate was significantly higher (67% vs. 38%, *P* < 0.05).

## 4 Discussion

In our study, we investigated the management of minimizing immunosuppressive therapy during an ICU stay for severe SARS-CoV-2 infection in 27 kidney transplant recipients, and their adequacy to local (French society of transplantation) or European (European renal association) expert recommendations (17).

An adequate management according to local quidelines was found for 63% of the patients upon admission, increasing to 85% during stay. A nephrological referral was immediately sought in 89% of cases. Immunosuppressive therapy was minimized in 100% of patients, but with various deviation from guidelines. Six patients (22%) did not comply with the recommendations, because the antimetabolite was reduced without discontinuation or reduced on admission and then discontinued later. The rate of antimetabolite discontinuation observed in our cohort (88%) is intermediate between that of the Turkish studies by Oto et al. (19) and Demir et al. (20) with 73% and 100% respectively. In a multicenter French study by Caillard et al. (21) including 279 kidney transplant patients with severe and non severe COVID-19, antimetabolites were stopped in 71.7% of cases, here too at the time of the recommendation issue.

Only one patient did not benefit from steroids increase. His infection occurred at the very beginning of the COVID-19 pandemic (March 2023), when very limited experience was available. The recommendations further suggested the use of 6 mg of dexamethasone daily. The adequate dosage was used 85% of patients in our cohort.

In parallel, European guidelines advocated for the discontinuation of all immunosuppressive drugs except steroids. However, continuing with low-dose CNI was to be considered for patients with higher risk of rejection (<1 year after transplantation and/or highly immunized). In our cohort, an adequate CNI adjustement was observed for 70% of cases upon admission and increasing to 85% during stay. In comparison, CNI were discontinued in only 57% of cases in the Demir et al. cohort (20), conducted early in the pandemic, which may explain the different management. Systematic CNI withdrawal was not advocated by the French guidelines, and discontinuation or tapering was be considered on an individual basis. CNIs were suspended in 52% of cases of severe COVID-19 in the French multicenter study (21), but should be interpreted causiously considering the inclusion period between March and April 2020.

Altogether, we found upon admission 63% of the patients to be in adequacy with the local guidelines and 59% to the European ones. In comparison, all treatments (CNI and antimetabolites) were discontinued in only 27% of cases in the Oto et al. cohort*.* (20). No significant association between the discontinuation of immunosuppressive drugs and the mortality was reported (22). In the French multicenter study (4), the mortality rate among all infected patients was 17.9% at 1 month and 23% at 2 months (23). However, the SARS-CoV-2 infection presented by their patients was less severe with only 13% of patients requiring vasopressors and 28.6% requiring mechanical ventilation. EER was used in 13.2% of cases. In our study, the ICU mortality was 44%, increasing up to 67% at one year post COVID-19, confirming the poor prognostic of this infection in kidney transplant recipients. However, the adequacy or inadequacy to either local or European guidelines was not associated with the one-year mortality.

Besides, immunosuppressive drugs with narrow therapeutic index such as CNI or mTOR inhibitors are to be closely monitored by trough level measurement. Only 39% of patients had an early trough level measurement, with a high risk of overdosing when 100% of patients with acute kidney dysfunction upon admission. It should also be noted that the mean trough levels for tacrolimus before ICU admission (8 ng/ml) was rather high, considering a median time of 3.7 years after transplantation.

Minimization could further expose the patient to the risk of rejection and graft loss to alloimmune injury. However, this hypothesis is controversial depending on the study, and the incidence of rejection after COVID-19 is variable (24). The largest study was carried out by Caillard et al. (21), in which 3.2% of SARS-CoV-2-infected KTR experienced graft loss. However the studies by Chen et al. (25) including 30 patients, or that by Elec et al. including 42 KTR (26), showed no rejection after reduction or even discontinuation of antimetabolites and CNI. In our cohort, no biopsy-proven acute rejection was observed in the aftermath of severe COVID-19. However, one patient lost his graft within six months post-transplant. In the absence of a biopsy, one can not discriminate between rejection and cortical necrosis. This variability between studies can be explained by the small number of patients or inadequate follow-up times.

Prevention of severe forms of COVID-19 disease in KTR relies on vaccination, even if the humoral response is lower, or the use of anti-SARS-CoV-2 monoclonal antibodies. In our work, we observed a trend towards a lower mortality rate in ICU and a significantly lower one-year mortality in vaccinated patients. Similarly, prophylactic monoclonal antibodies were shown to reduce mortality but did not reach significance in our small cohort. Of importance, the various types of monoclonal antibodies received by KTR and their adequacy to the actual SARS-CoV-2 variant identified for each patient could impact the outcome, but these points could not be addressed here given the small number of patients. This strategy is however currently being challenged and vaccination remain the corner stone of COVID-19 prevention. The use of curative monoclonal antibodies seemed to have little effect in our population, but we could not retrospectively asses the presence of counfounders in those patients (e.g., a greater severity upon admission). Those antibodies are no longer recommended as 1st-line treatment in the ICU.

Our study investigated the management of COVID-19 over a three-year period, which is longer than most of the above-mentionned studies, allowing a follow-up period of at least six months for all patients or for one year for all surviving patients. Nevertheless, this relatively long study period is associated with a significant improvement in knowledge and management of the disease. With this idea, we show (Figure 5D) a trend towards a lower mortality in the 2nd half of the study period. In a larger cohort, it would then be relevant to propose a sensitivity analysis of mortality risk factors, only in this most recent sub-population. Such a study was not possible in this work, given the small number of patients. We compared this COVID-19 group with a control group of KTR with a non-severe-COVID-19 ICU stay during the same period. Among possible risk factors for severe COVID-19, impairment in renal function 3 months prior to COVID-19 infection was the only statistical significance. As expected, the ICU stay characteristics were poorer in the COVID-19 group as well as the one-year death-censored graft survival, while the one-year mortality rate was significantly higher. The main limitations include the fact that it is a single-center study and the small number of patients, which limits the use of statistical models to study all mortality risk factors.

Overall, in this single-center cohort, the only variable associated with a reduction in mortality was vaccination, emphasizing that the key issue is immunization prior to infection, not restoration of immunity during ICU stay. Larger studies (transplant and non-transplant) have previously concluded that vaccination is the corner stone for prevention, lowering mortality and morbidity associated to COVID-19 disease. In the current context where the importance of vaccines is being questioned, we believe it is important to report locally on patients’ stories. We believe that our study illustrates the way in which patients were treated in the exceptional circumstances caused by SARS-Cov-2 virus, and could serve as a basis for reflection comparing the recommendations given by national and continental medical authorities and their actual application at a local level.

## Data Availability

The original contributions presented in the study are included in the article/Supplementary Material, further inquiries can be directed to the corresponding author.
