# Supplementary figures and images for: Adequacy to immunosuppression management guidelines in kidney transplant recipients with severe COVID-19 pneumonia: a practice survey
Source: Front Transplant. 2024 Mar 12;3:1305152. doi: 10.3389/frtra.2024.1305152 (PMC11235282; doi:10.3389/frtra.2024.1305152)

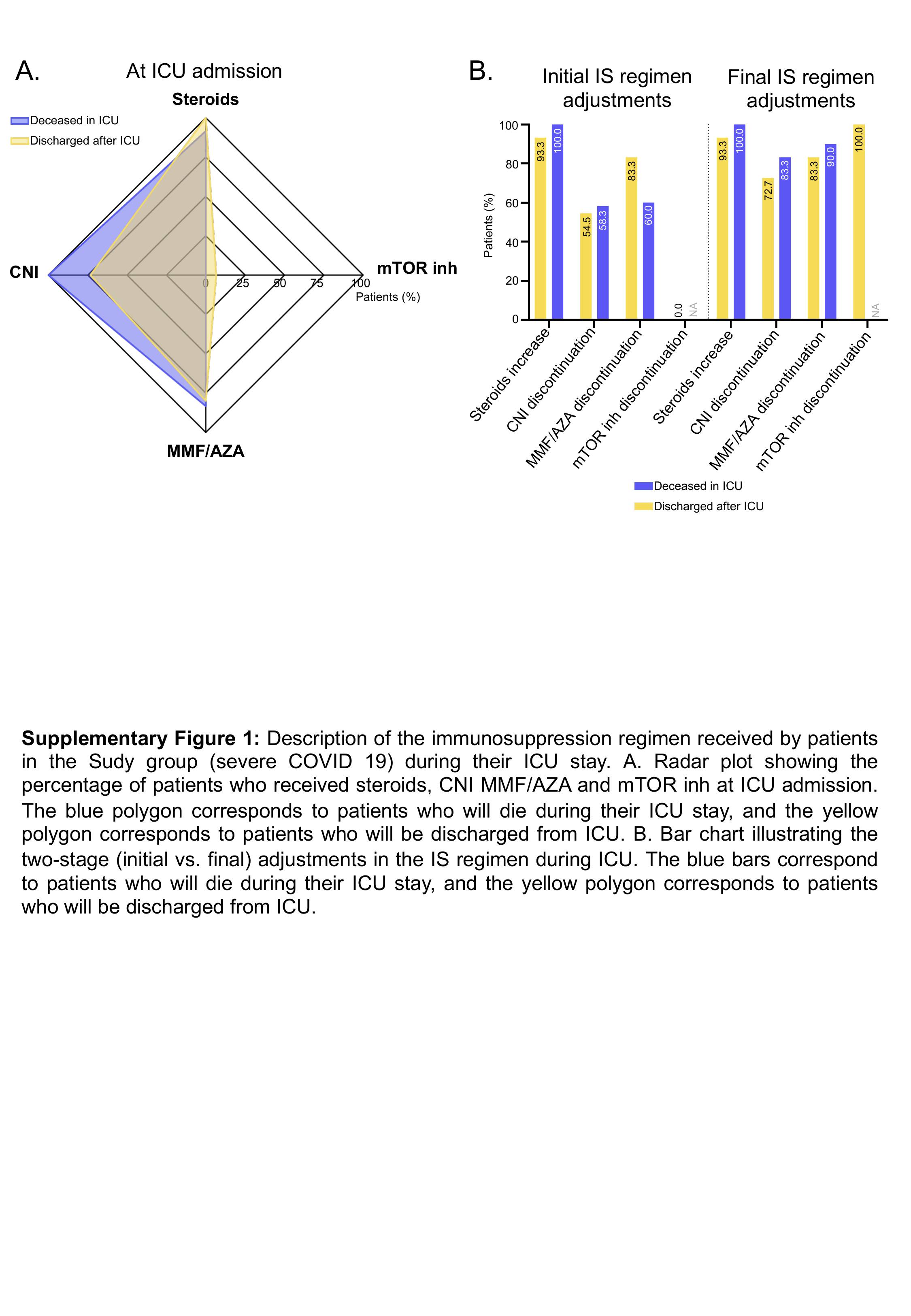

Supplement: Supplementary file 1 [file Image1.jpeg]

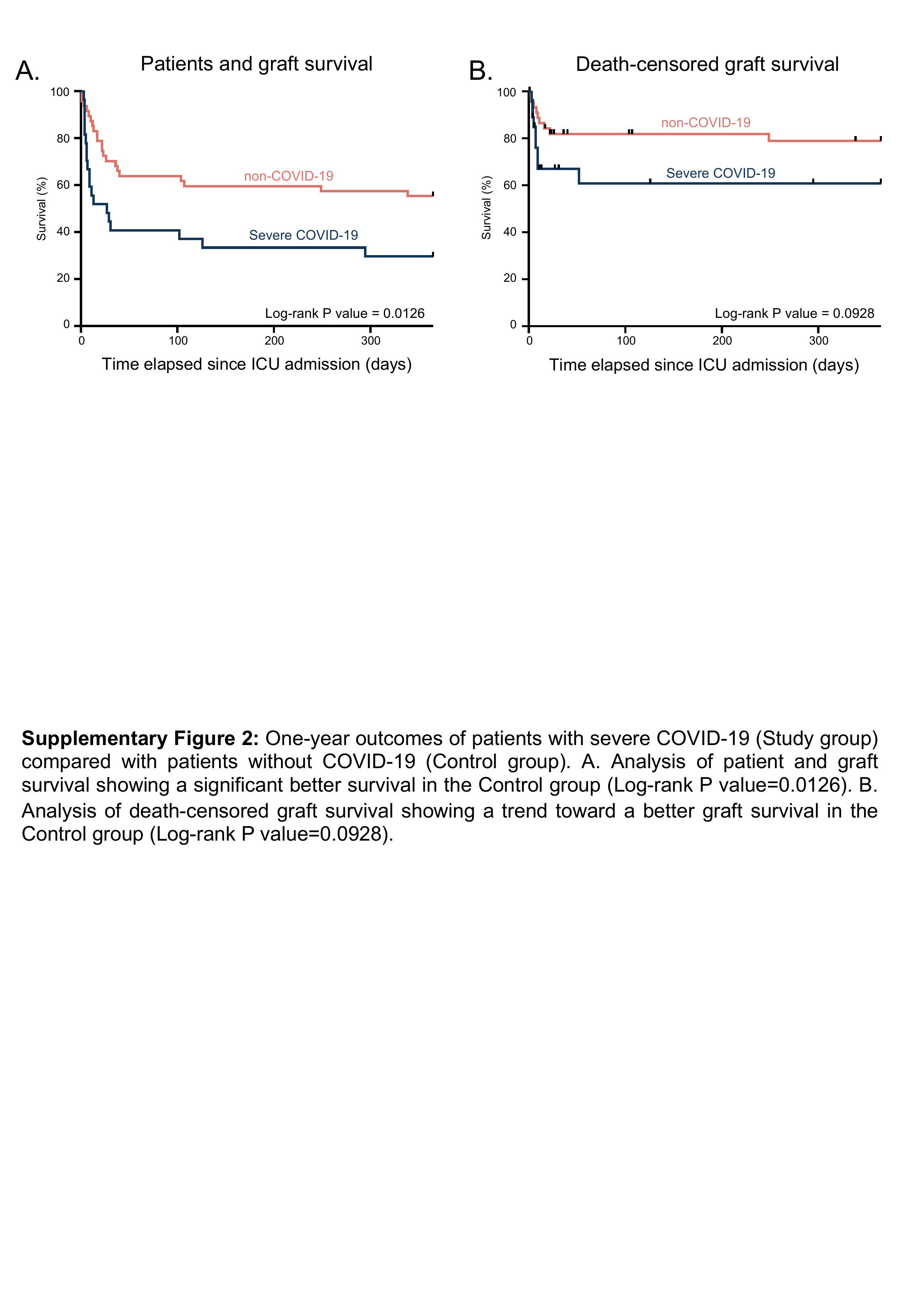

Supplement: Supplementary file 2 [file Image2.jpeg]
